# Advances in Hyaluronan Biology: Signaling, Regulation, and Disease Mechanisms

**DOI:** 10.1155/2015/690572

**Published:** 2015-09-13

**Authors:** Melanie A. Simpson, Carol de la Motte, Larry S. Sherman, Paul H. Weigel

**Affiliations:** ^1^Department of Biochemistry, University of Nebraska, Lincoln, NE 68588, USA; ^2^Department of Pathobiology, Cleveland Clinic Lerner Research Institute, Cleveland, OH 44195, USA; ^3^Division of Neuroscience, Oregon National Primate Research Center, Oregon Health & Science University, Beaverton, OR 97006, USA; ^4^Department of Biochemistry & Molecular Biology, University of Oklahoma Health Sciences Center, Oklahoma City, OK 73104, USA

Hyaluronan is an extracellular glycosaminoglycan polymer consisting of linear disaccharide units containing alternating glucuronate and N-acetylglucosamine. Many cell types make hyaluronan, which unlike most other macromolecules is assembled at the plasma membrane and concurrently translocated through the hyaluronan synthase enzyme. The normal function of large hyaluronan polymers (>1 MDa) in tissue cushioning, hydration, and lubrication is well established. The aberrant accumulation and degradation of hyaluronan and the receptor-mediated signaling of smaller hyaluronan fragments have also been extensively implicated in a variety of pathological states including inflammation and cancer. More recently, the discovery that hyaluronan can either be a structural matrix component or appear as smaller processed polymers and oligomers that differentially engage a diverse range of signaling receptors has created an exciting paradigm shift and reenergized hyaluronan research in a broad range of fields. In this special issue, eight review articles focus on summarizing the latest contributions to understanding hyaluronan synthesis and catabolism and the regulation of hyaluronan functions. Seven novel primary research articles also investigate multiple roles of hyaluronan in disease progression and targeting.

The review by P. H. Weigel discusses the mechanism of hyaluronan synthesis and polymer extrusion by the hyaluronan synthase family members as well as topological features of the enzymes, their functional requirement for associated lipids within the plasma membrane, and a proposed bioenergetic model for the concurrent translocation of hyaluronan to the extracellular space by the enzyme during synthesis. The review by M. Viola et al. addresses the regulation of hyaluronan synthesis by posttranslational modifications of HAS2 and the metabolic conditions that contribute to dysregulated synthesis in atherosclerosis. S. Shakya et al. review the recent data on cellular mechanisms such as autophagic release of hyaluronan-containing vesicles that are triggered in response to glucose overexposure and studies on the impact of altered hyaluronan synthesis in diabetic wound healing. J. M. Cyphert et al. provide an overview of hyaluronan synthesis and degradation, as well as a discussion of the widely differing signaling properties conferred by short processed oligomers versus long newly synthesized polymers of hyaluronan. L. S. Sherman et al. discuss the roles of hyaluronan in nervous system injury and propose a model by which the balance between hyaluronan synthesis and catabolism influences nervous system repair. M. E. Lauer et al. summarizes effects of environmental factors that stimulate hyaluronan production in the lung and review the functional studies that reveal a protective and regenerative role for hyaluronan polymers in lung injury repair. Finally, reviews by S. Misra et al. and S. Ghatak et al. summarize research on the interaction of hyaluronan with proteoglycans in the extracellular space and at the cell surface, in the context of wound healing and fibrosis, and discuss the potential for hyaluronan and proteoglycans to serve as therapeutic targeting agents in diverse disease states.

Novel research articles published in this special issue provide cutting edge methodology advances and insights into the mechanisms by which hyaluronan impacts cancer stem cells, facilitates cancer therapy, promotes inflammation, and controls immune function. The article by M. Shiina and L. Y. W. Bourguignon describes the characterization of microRNA expression induced by hyaluronan of different sizes in cancer stem cells and suggests that specific microRNA induction promotes cancer stem cell self-renewal upon exposure to high molecular mass hyaluronan. J. Woodman et al. provide a novel method for stabilizing cross-linked hyaluronan in nanoparticle construction and test the utility of these constructs for cytotoxic drug delivery in mouse xenografts. S. P. Kessler et al. show that HAS1/HAS3 null mice are less susceptible to chemically induced chronic inflammation than wild-type mice and do not develop colitis, indicating that hyaluronan production in the intestine, primarily by HAS3, is responsible for promoting chronic intestinal inflammation and inducing pathological changes in vasculature and leukocyte infiltration underlying colitis. The article by S. M. Ruppert et al. demonstrates a new role for hyaluronan signaling through CD44 in regulatory T cells undergoing development of resistance to cyclosporine A, independently of canonical IL-2 signaling. Research by T. Mirzapoiazova et al. examines the shedding of hyaluronan in exosomes and microvesicles, revealing that different sizes of hyaluronan are associated with these two very different vesicle types and that these vesicle types have opposite effects in disrupting or preserving vascular integrity. K. Rilla and A. Koistinen discuss the use of superresolution microscopy to visualize the induction of plasma membrane ruffling by HAS3-mediated hyaluronan synthesis. Finally, L. Do et al. describe gas-phase electrophoretic mobility molecular analysis, a novel method that permits highly sensitive size determination of hyaluronan in very small sample sizes.

Collectively, the results and perspectives in this special issue represent the latest description and summary of hyaluronan-mediated control mechanisms in normal and diseased tissues and highlight exciting research advances in a broad range of disease models that exploit novel chemistries and define paradigm-shifting concepts.

*Melanie A. Simpson* *Melanie A. Simpson* *Carol de la Motte* *Carol de la Motte* *Larry S. Sherman* *Larry S. Sherman* *Paul H. Weigel* *Paul H. Weigel*

